# The expression of Toll-like receptors in murine Müller cells, the glial cells in retina

**DOI:** 10.1007/s10072-012-1236-1

**Published:** 2012-12-04

**Authors:** Xiaomin Lin, Dan Fang, Hongyan Zhou, Shao Bo Su

**Affiliations:** Ocular Immunology Lab., The State Key Laboratory of Ophthalmology, Zhongshan Ophthalmic Center, Sun Yat-sen University, 54 S. Xianlie Road, Guangzhou, China

**Keywords:** Toll-like receptor, Müller Cell, Innate response, Cytokine, Eye

## Abstract

Müller cells, the principal glial cells of the retina, play an important role in immune responses. Toll-like receptors (TLRs) are members of the pattern recognition receptor family and mediate innate and adaptive immune responses. In this study, we isolated, characterized Müller cells from mouse retina, and analyzed the expression of TLRs in these cells. We found that the mRNA of TLR2, TLR3, TLR4, and TLR5 was highly expressed by Müller cells. PAM3 and LPS, the agonists for TLR2 and TLR4, promoted Müller cells to produce the inflammatory cytokine Interleukine-6 and the chemokine MIP-2/CXCL2. These results suggest that Müller cells may be involved in innate and adaptive responses via TLR signaling in the eye. Our study should facilitate further study of the role of Müller cell in eye diseases and identification of the potential therapeutic targets.

## Introduction

In mammalian retina, three types of glia have been identified: Muller glia, astroglia or astrocytes, and microglia. Müller cells constitute the main glial population in the retina [1]. They share a lineage with retinal neurons, and both Müller cells and neurons are derived from a common progenitor that is multipotent at all stages of retinal histogenesis [2]. They extend throughout the whole thickness of the neural retina, with nuclei in the inner nuclear layer and many fine processes surrounding neuronal cell bodies, axons, and blood vessels [3]. Müller cells subserve many of the metabolic, ionic, and extracellular buffering requirements of neurons [1]. Although much is yet to be learned about the functions of Müller cells in the retina, it is clear that they play important roles in retinal development, in the preservation of its integrity and in maintaining neuronal survival [4–6]. Thus, Müller cells constitute an anatomical link between the retinal neurons and the compartments to exchange molecules. This link is not merely anatomical but also functional. For this purpose, Müller cells are endowed with a wealth of different ion channels, ligand receptors, transmembrane transporter molecules, and enzymes [1, 7]. Many of these molecules are specifically expressed by Müller cells [8].

Toll-like receptors (TLRs) are a family of pattern recognition receptors which recognize distinct pathogen-associated molecular patterns (PAMPs), including molecules from bacteria, viruses, fungi, protozoa, and host cells [9–12]. Thus far, TLR1–10 in human and TLR1–9, 11–13 in mouse have been identified, and many of their ligands are known. For example, TLR2 is involved in the response to microbial lipoproteins and peptidoglycan, such as those in microbacteria, Gram-positive bacteria, and yeasts. TLR4 is required for the recognition of endotoxin of Gram-negative bacteria [11]. These receptors constitute the first line of defense against pathogens and play a crucial role in innate immune system by activating NF-*к*B and other signaling pathways to produce inflammatory cytokines [10, 11]. Furthermore, TLR signaling is also involved in the development of adaptive immune responses by upregulating costimulatory molecules of antigen presenting cells (APCs) [12]. Thus, TLRs play pivotal roles in innate and adaptive immune responses.

Several studies have shown the expression of TLRs in various cells in human central nervous system, including microglia, astrocytes, neurons, and vascular endothelial cell [13–15]. Since Müller cells constitute the main glial population of the retina, TLRs have ever been supposed to express in the Müller cells. Recent study showed that human Müller gail expressed all ten TLRs and the treatment by TLR ligands could enhance the expression of respective TLRs [14, 16]. The expression of TLR2, TLR3, and TLR4 has been detected activated retinal astrocytes isolated from B6 mice and also different TLR ligands had distinct stimulatory effects on these cells [17]. However, the expression of TLR and their responsiveness to TLR ligands in mouse retinal Müller cells remain to be investigated.

In this study, we established a stable Müller cell line from C57BL/6 mouse retina and determined their characteristics that provided a prerequisite for investigations on their properties. We found that TLR2, TLR3, TLR4, and TLR5 were expressed in murine Müller cells. We also investigated the activation of TLR2 and TLR4 in the Müller cells in response to the stimulation by respective ligand. The results showed that Müller cells stimulated by PAM3 and LPS, the agonists for TLR2 and TLR4, could markedly produce the inflammatory cytokine Interleukine-6 and the chemokine MIP-2/CXCL2. Our results suggest that Müller cells may play an important role in regulating innate and adaptive responses via TLR signaling in the eye.

## Materials and methods

### Experimental animals

Seven-day-old C57BL/6 mice were purchased from Laboratory Animal Center of Sun yat-sen University and approved by experimental animals monitoring station of Guangdong Province. Animal care and use were in compliance with institutional guidelines and with the Association for Research in Vision and Ophthalmology Statement for the Use of Animals in Ophthalmic and Vision Research.

### Tissue preparation, isolation, and culture of retinal Müller cells

Müller cells were isolated from mouse retina by collecting retinas from eight mice (7-day-old). First, eyes from the mice were collected and the connective tissue was removed. Then the eyes were immersed in serum-free DMEM on ice. Under a dissecting microscope, the anterior segment and vitreous were discarded. The neural retinas were rinsed with serum-free DMEM and collected by centrifugation at 400 g for 10 min and resuspended in complete medium (DMEM medium containing 10 % fetal bovine serum, 1 % l-glutamine and 1 % penicillin/streptomycin, Gibco). Then the retinal fragments were dissociated by gentle trituration through a Pasteur pipette and seeded on a single well of a six-well plate and incubated at 37° with 5 % CO_2_. Purification of cell cultures was obtained by repeating vigorous washing and removal of supernatant with non-attached cells (neuronal cells). Purity of primary culture was initially assessed by verification of homologous morphology using phase-contrast microscopy. When approaching confluence, cells were detached using 0.25 % trypsin/EDTA (Gibco) and subsequently split to obtain subcultures. Dissociated cells were cultured using complete medium at 37 ° with 5 % CO_2_. Müller cells were used in experiments after culture for 4–6 passages.

### Immunofluorescence assay

Müller cells were plated on fibronectin-coated glass coverslips and allowed to reach 70 % confluence. Cells were then rinsed twice with PBS, fixed with cold acetone for 10 min, and washed three times with PBS. The cells were then incubated with anti-mouse glutamine synthetase (GS) antibody (Abcam), CD11b antibody (BD Pharmingen) or isotype control at 4 °C overnight, respectively. Cells were washed three times with PBS on the second day and followed by incubation with a FITC-conjugated secondary antibody at 37 °C for an hour. Then cells were washed three times with PBS and Nuclei were stained with Hoechst (Sigma) for 30 s. The coverslips containing cells were mounted onto glass slides and photographed using a fluorescence microscope. Isotype control was analyzed under similar conditions.

### Real-time RT-PCR of TLR expression

Müller cells of passage 4 were seeded (5 × 10^5^ per well) in six-well plates. After cultured for 24 h, total RNA was extracted with TRIzol reagent (Invitrogen) according to the manufactuurer’s instructions. The cDNA was synthesized using PrimeScript RT reagent Kit (TaKaRa) in a final volume of 20 μL. The cDNA reactions were incubated at 37 °C for 15 min to synthesize and then 85 °C for 15 s to end. Real-time PCR assay was performed in ABI PRISM 7000, and the gene expression was examined by SYBR Green qPCR SuperMix-UDG (Invitrogen) with preincubation at 50 °C for 2 min and 95 °C for 2 min, and amplification of 40 cycles that was set for 15 s at 95 °C, and the annealing for 30 s at 60 °C. PCR amplification was performed in triplicate, and water was used to replace cDNA in each run as a negative control. The concentration of the gene of interest was determined using the comparative threshold cycle (CT) number and normalized to that of the GAPDH (amount of target = 2^−∆CT ^%). The mouse GAPDH, TLR1–9, and TLR11–13 specific primers used in reaction were listed in Table 1.

**Table 1:** Primer sequences of mouse TLRs and GAPDH for Real-time RT-PCR

Gene	Sequence (5′ → 3′)	Accession number	Size (bp)
GAPDH	F:TGAGCAAGAGAGGCCCTATC R:AGGCCCCTCCTGTTATTATG	NM_008084.2	94
TLR1	F:TCAAGTGTGCAGCTGATTGC R:TAGTGCTGACGGACACATCC	NM_030682.1	178
TLR2	F:CAGCTGGAGAACTCTGACCC R:CAAAGAGCCTGAAGTGGGAG	NM_011905.3	193
TLR3	F:CCTCCAACTGTCTACCAGTTCC R:GCCTGGCTAAGTTATTGTGC	NM_126166.4	230
TLR4	F:CAACATCATCCAGGAAGGC R:GAAGGCGATACAATTCCACC	NM_021297.2	206
TLR5	F:AGCATTCTCATCGTGGTGG R:AATGGTTGCTATGGTTCGC	NM_016928.2	219
TLR6	F:TGGATGTCTCACACAATCGG R:GCAGCTTAGATGCAAGTGAGC	NM_011604.3	206
TLR7	F:TTCCTTCCGTAGGCTGAACC R:GTAAGCTGGATGGCAGATCC	NM_133211.3	203
TLR8	F:TCTACTTGGCCTTGCAGAGG R:ATGGCAGAGTCGTGACTTCC	NM_133212.2	273
TLR9	F:CAAGAACCTGGTGTCACTGC R:TGCGATTGTCTGACAAGTCC	NM_031178.2	200
TLR11	F:TCCTTCCTCTGATTAGCTGTCCTAA R:TCCACATAATTTCCACCAACAAGT	NM_205819.2	86
TLR12	F:GCCGCCATTCCAAGCTATC R:CTCCACAGTCCGAGGTACAACTT	NM_205823.2	89
TLR13	F:ATGGCACAAAACGGAGAAGAA R:CTTTGTATACCCATGCCTCATCAG	NM_205820.1	80

### Analysis of cytokine secretion by stimulated Müller cells with PAM3 and LPS

Müller cells of passage 6 were seeded (5 × 10^5^ per well) in six-well plates. After being cultured for 24 h, Müller cell monolayer was incubated in the presence of PAM3 (1 μg/ml) or LPS (0.1 μg/ml), or PBS control. After 6 h, cells were collected and total RNA was extracted with TRIzol reagent. Real-time RT-PCR assays were performed as described earlier to determine mRNA expression of inflammatory cytokines and chemokines including Interleukine-6 (IL-6), Interleukine-1β (IL-1β), Interleukine-10 (IL-10), Tumor necrosis factor-α (TNF-α), MIP-2/CXCL2, MCP-1/CCL2, and MIP-1α/CCL3. The specific primers’ sequences are shown in Table 2. PCR amplification was performed in triplicate, and water was used to replace cDNA in each run as a negative control. The CT method normalized to GAPDH was used to analyze relative changes in gene expression (amount of target = 2^−∆∆CT^). To determine the protein production of cytokines and chemokines released by stimulated Müller cell, 5 × 10^5^ cells were seeded in a well of six-well plates. After being cultured for 24 h, cells were, respectively, challenged with TLR2 and TLR4 ligand, PAM3 (1 μg/ml) and LPS (0.1 μg/ml) for another 24, 48, and 72 h. Müller cells cultured in the absence of TLR ligand were included as controls. Then culture media were collected and ELISA used for measurement of inflammatory cytokines and chemokines. ELISAs were performed according to the manufacturer’s instructions (R&D systems, Minneapolis, MN). The amount of cytokines in the culture media was expressed as pictograms per milliliter media.

**Table 2:** Primer sequences of inflammatory cytokines and chemokines for Real-time RT-PCR

Gene	Sequence (5′ → 3′)	Accession number	Size (bp)
IL-6	F:CCAGAGATACAAAGAAATGATGG R:ACTCCAGAAGACCAGAGGAAAT	NM_031168.1	88
IL-1β	F:ACACTCCTTAGTCCTCGGCCA R:TGGTTTCTTGTGACCCTGAGC	NM_008361.3	51
IL-10	F:GGTTGCCAAGCCTTATCGGA R:ACCTGCTCCACTGCCTTGCT	NM_010548.2	191
TNF-α	F:GCACCACCATCAAGGACTCA R:TCGAGGCTCCAGTGAATTCG	NM_013693.2	51
MIP-2	F:CGCCCAGACAGAAGTCATAG R:TCCTCCTTTCCAGGTCAGTTA	NM_009140.2	132
MCP-1	F:TAGGCTGGAGAGCTACAAGAGG R:AGTGCTTGAGGTGGTTGTGG	NM_011333.3	277
MIP-1α	F:TGAATGCCTGAGAGTCTTGG R:TTGGCAGCAAACAGCTTATC	NM_011337.2	134

## Statistics

Experiments were repeated three times. Results were highly reproducible. Figures show pooled data from repeat experiments, or representative experiments, as indicated. The statistical analysis was performed using an independent *t* test. *p* values less than 0.05 were considered significant.

## Results

### Isolation and culture of Müller cells

**Fig. 1:**
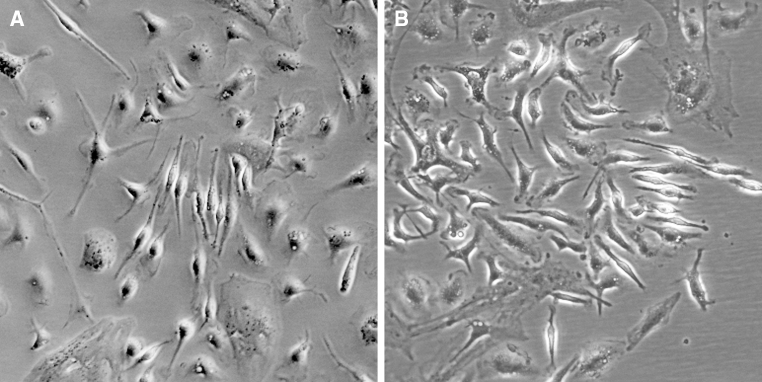
Morphology of cultured Müller cells, which were photographed using a phase-contrast microscope in a digital format (×100). **a** Cultured cells on day 10 displayed the Müller cell features comprising a characteristic bipolar, spindle-shape morphology, cytoplasmatic projections, a central nucleus, and an elongated shape. **b** Primary Müller cells were approaching a confluent monolayer on day 14 of culture and adopted a fibroblast-like morphology, and became increasingly flattened

Primary cultures of dissociated retinal cells appeared heterogeneous after 3 days of incubation. To characterize cultured cells, phase-contrast microscopy was used to assess morphological features. Besides single cells and debris, non-adherent cells forming cellular clusters were observed. By gentle panning of the culture plate, some cells were attached to the dish. By day 5, non-confluent cells started to form bipolar extensions exhibiting a flat and elongated shape. On the 7th day, the culture supernatant and non-adherent debris were removed and the cells were vigorously washed, leaving single, asymmetrical adherent cells. Forming of projections increased within the next 48–72 h of culture resulting in pronounced Müller cell features comprising a characteristic bipolar morphology, cytoplasmatic projections, and an elongated shape (Fig. 1a). At this stage, the cells acquired the ability to proliferate. When approaching a confluent monolayer, cells of the primary cultures adopted a fibroblast-like morphology and became increasingly flattened (Fig. 1b). After 14 days, when reaching confluence, the primary cell culture was divided into subcultures by incubation with 0.25 % trypsin/EDTA. Then dissociated cells were plated in a 25-cm^2^ flask and cultured with complete DMEM in an incubator at 37 °C with 5 % CO_2_. Most subculture of cells were evenly distributed and attached to the flask within 24 h of incubation. These cells proliferated rapidly, approaching a confluent monolayer in 2–3 days. All passaged cells exhibited the same bipolar and elongated morphology with several cytoplasmic projections.

### Immunofluorescence assay on Müller cells

**Fig. 2:**
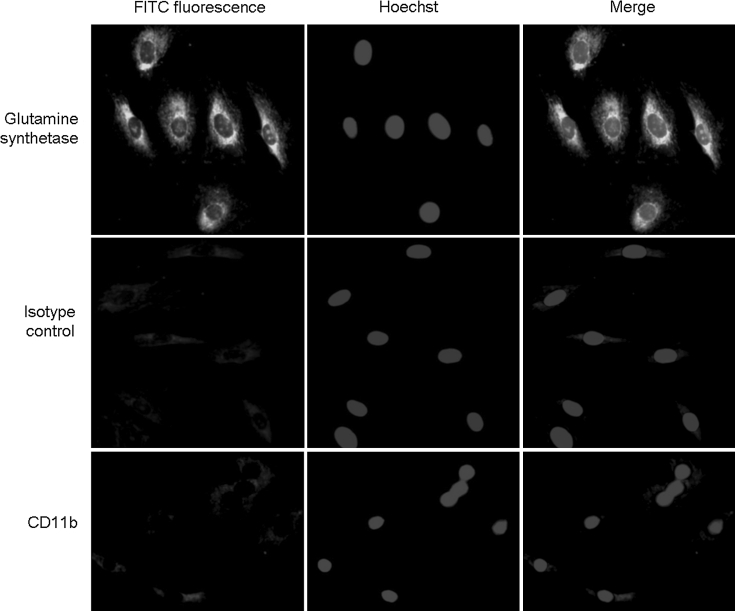
Immunofluorescence staining of GS on passage 4 Müller cells. *Upper panel* cells stained with rabbit anti-GS antibody (*green*). The expression of GS appeared predominantly around the central nuclei (*blue*). *Middle panel* Müller cells stained with isotype control antibody. *Lower panel* Müller cells stained with anti-cd11b antibody

It was reported that glutamine synthetase (GS) was a specific marker for Müller cells [18, 19]. However, recent reports showed that microglial cells in the brain also expressed GS [20]. Additional studies showed that microglial cells, but not Müller cells, expressed CD11b [21–23]. To determine the purity and exclude contamination of Müller cell preparations by microglial cells and further characterize the cultured cells, we performed immunoflurescence assay to stain isolated cells at passage 4. Cells grown on glass slides were stained with mouse GS and anti-CD11b antibodies. The result showed that GS was detected in all tested cells at passage 4. The expression was in a punctuated pattern that appeared predominantly around the central nuclei and less in cell projections (Fig. 2). The cells were negative in isotype control or anti-CD11b antibody staining (Fig. 2). These results indicate that all the cells examined were Müller cells rather than microglial cells.

### TLR mRNA expression in Müller cells

**Fig. 3:**
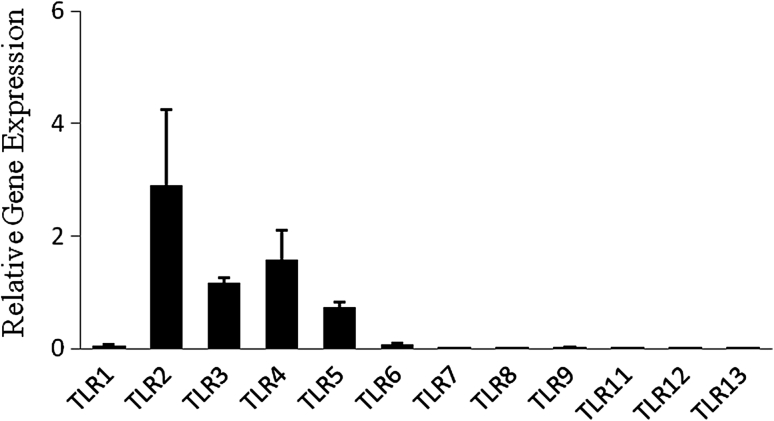
Real-time PCR analysis of TLR expression in Müller cells. Total RNA was extracted from passage 4 Müller cells and cDNA was generated. The expression of TLRs was analyzed by real-time PCR using specific primers. The concentration of the genes of interest was determined using the comparative threshold cycle number and normalized to that of the GAPDH (amount of target = 2^−∆CT ^%). Representative results from three independent experiments are shown

Müller cells isolated from the retina of C57BL/6 mice at 4 passages as described as above were used. Total mRNA was extracted from the cells and real-time RT-PCR assay was used to determine the expression of TLR mRNA. Data showed that TLR2, TLR3, TLR4, and TLR5 mRNA were highly expressed in Müller cells (Fig. 3). TLR1 and TLR6, but not TLR7, TLR8, TLR9, TLR11, TLR12, and TLR13, were also detected in Müller cells (Fig. 3).

### The expression of cytokines and chemokines by TLR ligand-stimulated Müller cells

**Fig. 4:**
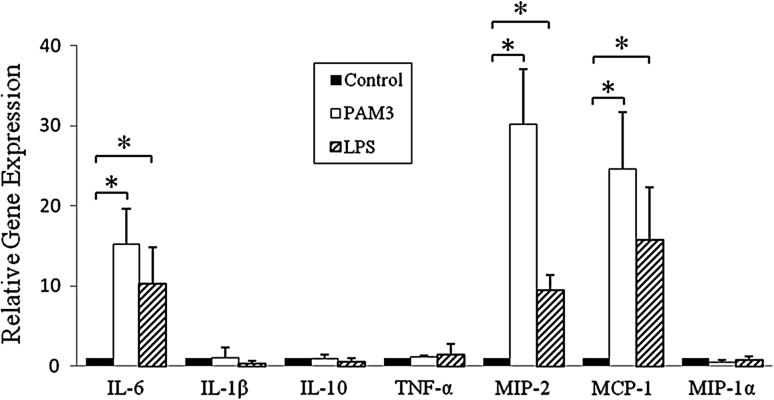
Cytokine and chemokine expression by TLR2 or TLR4-ligand stimulated Müller cells. Total RNA was isolated from passage 6 Müller cells which were treated with PAM3 (1 μg/ml) or LPS (0.1 μg/ml) for 6 h. cDNA was generated and the expression of IL-6, IL-1β, IL-10, TNF-α, MIP-2/CXCL2, MCP-1/CCL2, and MIP-1α/CCL3 was analyzed by real-time PCR using specific primers. The concentration of the gene of interest was determined using the comparative threshold cycle number and normalized to that of the GAPDH (amount of target = 2^−∆∆CT^). Representative results from three independent experiments are shown. All values represent the mean ± SEM. *p < 0.05, statistically significant difference in cytokine and chemokine expression from the control group

Our above results showed that TLR2 and TLR4 were highly expressed in Müller cells. To determine the function of TLR2 and TLR4 on Müller cells, the cells were cultured with TLR ligands (PAM3 and LPS) for 6 h. Total RNA was isolated and real-time RT-PCR assays were performed. The results showed that the mRNA expression of IL-6, MIP-2/CXCL2,and MCP-1/CCL2 in stimulated Müller cells were significantly increased compared with control cells (Fig. 4). In contrast, the expression of IL-1β, IL-10, TNF-α, and MIP-1α/CCL3 had no significant change in the Müller cells which were treated with TLR2 and TLR4 ligands (Fig. 4).

### Increased release of IL-6 and MIP-2/CXCL2 by TLR2 or TLR4 ligand-stimulated Müller cells

**Fig. 5:**
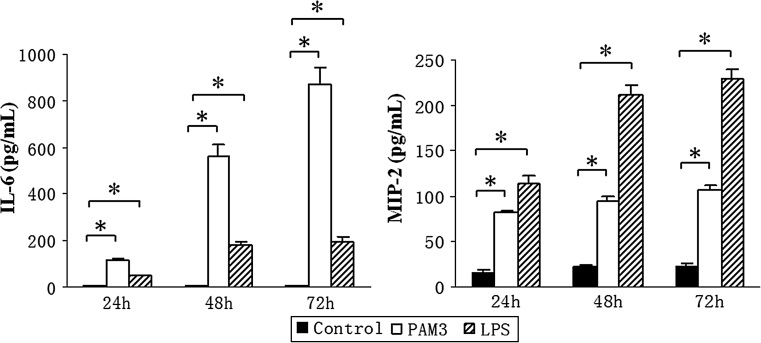
The production of IL-6 and MIP-2/CXCL2 by PAM3- or LPS- stimulated Müller cells. Müller cells were stimulated with PAM3 (1 μg/ml) or LPS (0.1 μg/ml). The supernatants were collected at 24, 48, or 72 h after treatment. Cytokine production was measured by ELISA. Representative results from three independent experiments are shown. The values represent the mean ± SEM. *p < 0.05, statistically significant difference in cytokine production from the control group

As the mRNA expression of IL-6 and MIP-2/CXCL2 were significantly increased in PAM3- and LPS- treated Müller cells, ELISA was used to measure the production of IL-6 and MIP-2/CXCL2 in the supernatants of PAM3- and LPS- stimulated Müller cells. In accordance with the real-time RT-PCR results, the ELISA results showed that the production of IL-6 and MIP-2/CXCL2 was markedly increased by PAM3 and LPS-stimulated Müller cells (Fig. 5). Our results thus indicate that Müller cells expressed functional TLR2 and TLR4 which play an important role in response to stimulation by microbial molecular patterns as well as potential endogenous ligands in the eye.

## Discussion

In this study, we described a method for isolation and propagation of Müller cells from murine retina. We successfully isolated and characterized these cells and established a stable murine Müller cell line, which provides a resource for further investigations on murine Müller cell physiology and subsequent studies to elucidate their participation in most of the signaling highway and immune responses. Many different methods were previously used to isolate Müller cells from the retina of rat, rabbit, porcine, and equine [24–27]. In these methods, Müller cells were dissociated from enzyme-digested retina and purified using sequential density gradient centrifugation. Because Müller cells have the ability to migrate from the retina, adhere to plastics and proliferate in vitro [8], in our study, we successfully isolated and expanded Müller cells by a method in which retina was triturated into small pieces without enzyme treatment and cells obtained were cultured in complete medium.

Müller cells are often identified by morphology and specific markers. In our study, the morphology of Müller cells isolated from mouse retinas was similar to the morphology of Müller cells from human, rat, cat, rabbit, and guinea pig [26–31]. Previous studies showed that glutamine synthetase (GS) was a specific marker and exclusively present in Müller cells. However, recent studies showed that microglial cells in the brain also expressed GS. Therefore, we stained isolated Müller cells with the antibody against mouse CD11b, a positive surface marker for microglia, but not Müller cells. Our results indicate that all our isolated cells were Müller cells rather than microglial cells.

The discovery of mammalian TLRs has provided new insights into the association between innate responses to microbes and adaptive responses and consequently their impact on autoimmune diseases. TLRs recognize microbial as well as endogenous ligands from necrotic cells to induce inflammatory responses [32]. Many observations suggest a role of TLRs in triggering allergic and autoimmune diseases [33, 34]. On the other hand, unexpected observations have indicated that systemic TLR stimulation can prevent the onset of allergic and autoimmune diseases when it is implemented early enough in the natural history of the disease [35, 36]. Recent study showed that TLR2 played an important role in mediating innate response to *S. aureus* by Muller glia [37]. Our previous studies showed that signaling of TLR2, TLR3, TLR4, and TLR9 is highly redundant in the adjuvant effect needed to induce experimental autoimmune uveitis (EAU) [38, 39]. Our recent study suggest that activation of innate immune system mediated by TLR3 signaling pathway is of importance in the pathogenesis of virus-induced autoimmune diseases [40]. In this study, we found that TLR2, TLR3, TLR4, and TLR5 were highly expressed in Müller cells. Our study also indicated that Müller cells expressed functional TLR2 and TLR4. These results suggest that Müller cells may be involved in endophthalmitis through TLR2 and TLR4 signaling. Our study will facilitate study of Müller cells in intraocular immune responses in endophthalmitis and to explore the potential targets for therapeutic agent.
